# The relationship between perceived outdoor sports atmosphere and social-emotional competence among college students: the mediating role of core self-evaluation and the moderating role of digital health literacy

**DOI:** 10.3389/fpsyg.2026.1947247

**Published:** 2026-10-28

**Authors:** Junhong Cui, Namei Song, Xiaobei Gu, Shawei Zhang

**Affiliations:** 1 Chongqing College of International Business and Economics, Hechuan, Chongqing, China; 2 School of Physical Education and Health Sciences, Chongqing Normal University, Shapingba, Chongqing, China

**Keywords:** college students, core self-evaluation, digital health literacy, outdoor sports atmosphere, social-emotional competence

## Abstract

**Objective:**

This study aims to explore the relationship between the perceived outdoor sports atmosphere and social-emotional competencies among college students, with a focus on examining the mediating role of core self-evaluation and the moderating role of digital health literacy, to provide empirical evidence for understanding the psychosocial mechanisms underlying the development of social-emotional competencies among college students.

**Methods:**

Using convenience sampling, we selected 1,218 college students in Chongqing as survey participants. The survey utilized the Outdoor Sports Atmosphere Scale, the Core Self-Evaluation Scale, the Digital Health Literacy Scale, and the Social-Emotional Competence Scale. We analyzed the data using SPSS 27.0 and the PROCESS 4.1 plugin. The significance of the mediating and moderating roles was tested using the bias-corrected Bootstrap method (with 5,000 resampling iterations).

**Results:**

The perceived outdoor sports atmosphere was significantly positively associated with social-emotional competence (β = 0.232, *p* < 0.001). Core self-evaluation showed a significant indirect association with the relationship between the perceived outdoor sports atmosphere and social-emotional competence (indirect effect = 0.124, 95% CI [0.100, 0.150]), accounting for 34.73% of the total effect. Digital health literacy positively moderates the relationship between the perceived outdoor sports atmosphere and core self-evaluation (interaction term β = 0.135, *p* < 0.001); the higher the level of digital health literacy, the stronger the positive association of the perceived outdoor sports atmosphere with core self-evaluation, thereby indirectly associated with higher social-emotional competence.

**Conclusion:**

Perceived outdoor sports atmosphere was positively associated with social-emotional competence, both directly and indirectly via core self-evaluation; furthermore, digital health literacy, as a moderating factor, is linked to a stronger positive effect of the perceived outdoor sports atmosphere on core self-evaluation. These findings expand our understanding of the cognitive pathways linking the perceived outdoor sports atmosphere to college students' social-emotional competencies and the individual literacy conditions on which these pathways depend, providing valuable insights for promoting college students' participation in outdoor sports and enhancing their social-emotional competencies.

## 1 Introduction

In response to rapid changes in the global labor market, educational reforms around the world are placing increasing emphasis on cultivating students' higher-order thinking and complex problem-solving skills. Enhancing college students' social-emotional competence (SEC) has become a key strategic issue in international educational development. According to CASEL's definition, SEC refers to the process by which individuals acquire and apply relevant knowledge, skills, and attitudes to shape a healthy sense of self, manage emotions, achieve personal and collective goals, express empathy, build and maintain supportive relationships, and make responsible and empathetic decisions (CASEL, 2020). Both the Organization for Economic Cooperation and Development and the World Bank emphasize that SEC plays a critical role in individual development, mental health, and career success(OECD, 2015; World Bank, 2019). A wealth of research confirms that SEC is significantly linked to higher students' academic performance (Corcoran et al., 2018), well being (Taylor et al., 2017), and mental health (Shum et al., 2025). The college years mark a critical transition from adolescence to early adulthood. During this period, students must adapt to a new social and academic environment and face numerous challenges in adjusting their learning styles, interpersonal relationships, and social roles. SEC serves as an important protective factor during this transition. Therefore, an in-depth exploration of the determinants and mechanisms of SEC among college students holds significant theoretical value and practical importance.

Existing research indicates that students' SEC is influenced by factors such as teacher-student relationships (Li et al., 2026; Ma et al., 2026), family factors (Wen et al., 2026), psychological resilience, and self-efficacy (Wang et al., 2026). In the field of health promotion, outdoor playtime (Ferguson et al., 2025), dance instruction (Xu and Liu, 2026), physical exercise (Cao et al., 2026), and physical activity (Chen et al., 2026; Zhao et al., 2026) have all been shown to enhance SEC. However, most of these studies have focused on the individual behavioral level, with relatively little attention paid to perceptual factors at the physical activity environment level. In this paper, “outdoor sports atmosphere” refers to an individual's subjective perception in an outdoor sports setting. Specifically, it measures students' subjective perceptions across the following five dimensions: interpersonal connection, nature connection, interpersonal barriers, information access, and conditional barriers. It is a perceived construct rather than an objective measure of campus facilities, institutional resources, green-space availability, or other campus characteristics. It is important to conceptually distinguish perceived outdoor sports atmosphere from actual physical activity participation. The former is a perceived environmental construct. The latter is a behavioral construct. These two constructs are conceptually distinct and may operate through different pathways to influence psychosocial outcomes.

Recent research on affective exercise experiences among Chinese college students has demonstrated that subjective and affective experiences surrounding exercise—such as pleasure, enjoyment, and emotional engagement—constitute a measurable construct distinct from exercise behavior itself (Wang et al., 2023). This work underscores the importance of examining perceived and experiential dimensions of the exercise environment rather than treating them as proxies for actual behavior. Building on this conceptual foundation, the present study focuses on perceived outdoor sports atmosphere as a distinct environmental perception construct, rather than as an indicator of actual physical activity participation. Existing research indicates that a positive exercise atmosphere can enhance exercise persistence (Liu et al., 2011). Currently, there is a lack of sufficient empirical testing regarding the relationship and underlying mechanisms between the perceived outdoor sports atmosphere and social-emotional competence among college students, and further exploration is urgently needed.

### 1.1 The perceived outdoor sports atmosphere and social-emotional competence

As a comprehensive activity that integrates physical activity, nature experiences, and social interaction, outdoor sports have a positive impact on individuals' physical and mental health. Existing research has shown that water-based outdoor sports activities have a significant impact on college students' mental health (Liu x. et al., 2025), and outdoor adventure education may also help enhance adolescents' interpersonal connections and psychological strengths (Down et al., 2025). A systematic review further indicates that activities such as hiking, cycling, outdoor camping, and nature exploration are closely associated with reduced anxiety and depression, improved mood, strengthened social connections, and enhanced environmental responsibility (Vasilaki et al., 2025). A study of a multiethnic urban cohort found that time spent playing outdoors was positively correlated with children's social-emotional competencies (Ferguson et al., 2025). These studies suggest that outdoor physical activity may contribute to the development of individuals' social-emotional competencies by fulfilling basic psychological needs, reducing stress, fostering interpersonal connections, and promoting self-concept restructuring. Furthermore, the relationship between physical exercise and social-emotional competencies has also attracted widespread attention from researchers. Existing research has found that physical exercise is not only directly associated with an individual's social adaptability (Liu et al., 2023) but is also significantly positively correlated with social-emotional competencies (Cao et al., 2026; Wang et al., 2026). It is worth noting that the benefits individuals derive from outdoor physical activity do not depend solely on the exercise behavior itself but are also influenced by their perceived environment. According to ecosystem theory (Bronfenbrenner, 1979), an individual's development is shaped by the interactions of multiple environmental systems, among which the school—as a key component of the microsystem—plays a crucial role in students' psychosocial development. Compared to physical activity at the individual level, the perceived environment may trigger psychological processes at an earlier stage.

Conceptually, the outdoor sports atmosphere captures individuals' subjective evaluations of the environment, which may influence social-emotional competence through experiential and symbolic processes that are independent of actual physical activity participation. For instance, merely perceiving a supportive environment can foster a sense of belonging and emotional security, and perceived norms can shape self-concept even among non-participants. This distinction aligns with research on affective exercise experiences, which shows that subjective experiences of exercise are distinct from exercise behavior itself and can independently predict physical activity (Wang et al., 2023). Therefore, it is necessary to examine the relationship between perceived outdoor sports atmosphere and social-emotional competence, as well as its underlying mechanisms, from an environmental perception perspective.

### 1.2 The mediating role of core self-evaluation

Core self-evaluation refers to an individual's fundamental assessment of their self-worth, abilities, and sense of control, and consists of four core components: self-esteem, general self-efficacy, neuroticism (emotional stability), and locus of control (Judge et al., 2003). According to social cognitive theory (Bandura, 1986), environmental factors indirectly influence an individual's behavior and psychological development by affecting their cognitive evaluation system. This provides a theoretical basis for understanding the mediating pathways of core self-evaluation.

As a core dimension of college students' perception of their physical activity environment, perceived outdoor sports atmosphere may be associated with students' core self-evaluation by providing experiences of mastery, social modeling, and positive feedback. Previous research has shown that extracurricular physical exercise is significantly positively correlated with core self-evaluation (Zhu et al., 2024); physical exercise can also indirectly alleviate depressive symptoms through core self-evaluation (Ji et al., 2026). Research on affective exercise experiences further indicates that subjective emotional experiences during exercise are distinct from exercise behavior itself and can independently predict physical activity (Wang et al., 2023). A positive sports atmosphere is also associated with psychological resilience and a growth mindset among college students (Zhang and Li, 2025), as well as with enjoyment and exercise self-efficacy (Zhang et al., 2025a), which may serve as intermediate processes linking exercise atmosphere to behavioral outcomes. Intervention studies have also shown that structured mind-body exercise can reduce burnout and stress among female undergraduate students (Zhu et al., 2021), suggesting that exercise-related experiences may contribute to psychological resource accumulation in university populations. Individuals with higher core self-evaluation typically exhibit greater emotional stability, higher levels of optimism, and more effective interpersonal coping strategies—all of which are core elements of social-emotional competence. Existing research indicates that core self-evaluation is significantly positively correlated with interpersonal adaptability (Zhang et al., 2025b) and social adaptability (Wang et al., 2025). Furthermore, psychological resilience (Xu and Liu, 2026) and self-identity (Wu et al., 2026) have also been shown to positively predict social-emotional competence. These findings suggest that core self-evaluation may serve as a cognitive bridge linking environmental factors to social-emotional competence.

### 1.3 The moderating role of digital health literacy

Digital health literacy refers to an individual's ability to use digital technologies to access, understand, evaluate, and apply health information to maintain physical and mental well being (Norman and Skinner, 2006). In the digital age, DHL has become a key literacy that is related to individuals' health decisions and behaviors. Existing research indicates that DHL is significantly associated with college students' physical exercise behaviors (Zhou N. et al., 2025) and health self-management behaviors (Zhou Y. et al., 2025), and that DHL is significantly positively correlated with psychological resilience (Liu et al., 2024) and self-efficacy (Yao et al., 2026). These findings suggest that DHL is not only foundational for processing health information but also an important precursor to mobilizing psychological resources and engaging in health-promoting behaviors.

Regarding the moderating role of DHL, prior research has shown that DHL moderates the relationship between physical exercise atmosphere and sleep quality (Li, 2024), and between self-efficacy and health knowledge(Prinitha and Jose, 2025). This suggests that DHL may function as a boundary condition between environmental factors and psychological outcomes. Accordingly, DHL is expected to moderate the first stage of the mediation model. Taken together, these theoretical perspectives provide an integrated explanation for the proposed model. Ecological systems theory (Bronfenbrenner, 1979) identifies the school outdoor sports atmosphere as an environmental resource; social cognitive theory (Bandura, 1986) specifies that this resource is mediated by cognitive evaluation (core self-evaluation); and conservation of resources theory (Hobfoll, 2002) suggests that individuals with greater initial resources (e.g., DHL) are better able to transform environmental resources into psychological resources. This integrated logic clarifies why DHL should moderate the first stage of the mediation model—the link between perceived outdoor sports atmosphere and core self-evaluation—rather than the direct path to social-emotional competence. Prior research has shown that individual difference factors can moderate the relationship between cognitive appraisals of physical activity and behavioral outcomes (Chen J. et al., 2023), supporting the broader argument that individual characteristics can condition exercise-related processes. Therefore, this paper proposes the following hypothesis (see Figure 1).

**Figure 1:**
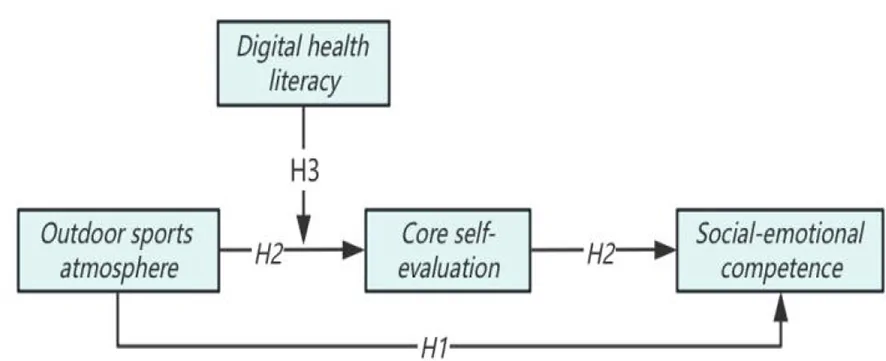
Hypothetical model diagram.

- H1: Perceived outdoor sports atmosphere is positively associated with college students' social-emotional competence.
- H2: Core self-evaluation shows a significant indirect association between the perceived outdoor sports atmosphere and social-emotional competencies.
- H3: Digital health literacy positively moderates the relationship between the perceived outdoor sports atmosphere and core self-evaluation.
- H4: Digital health literacy moderates the indirect effect of core self-evaluation on the relationship between the perceived outdoor sports atmosphere and social-emotional competencies.

## 2 Materials and methods

### 2.1 Participants and study design

This study employed convenience sampling, selecting university students (including undergraduate and graduate students) at regular universities in Chongqing, China, as the survey participants. The sample included four universities: Chongqing Normal University (teacher education), Southwest University (comprehensive), Chongqing University of Technology (science and engineering), and Chongqing University of International Business and Economics (economics and finance). Participants were recruited online via the “Wenjuanxing” platform; survey links were shared by student advisors in the WeChat or QQ groups for the classes they oversee, and students completed and submitted the questionnaires anonymously on a voluntary basis. The survey was conducted from June to July 2026. Prior to completing the questionnaire, researchers explained to participants the purpose of the study, the anonymity of their responses, the voluntary nature of participation, and their right to withdraw from the study at any time. The system was configured to require participants to check the electronic option “I agree to participate” before accessing the main questionnaire; all participants signed an informed consent form online. This research protocol has been reviewed and approved by the Ethics Committee of Chongqing University of International Business and Economics (Approval No.: XSWYH2026001). All data were anonymized, collected, and stored in accordance with the ethical principles of the Declaration of Helsinki.

We used two complementary approaches to justify our sample size. First, a precision-based estimate was computed using PASS 23.0 to determine the minimum sample size required for estimating population parameters with adequate precision. The confidence level was set at 1-α = 0.95 and the confidence interval width (two-tailed) at 0.06, yielding a minimum sample size of N = 1,098. Accounting for a 20% nonresponse rate, the target sample size was set at no less than 1,373. This precision-based approach is a conservative strategy commonly used in cross-sectional survey research (Cochran, 1977). Second, to ensure adequate statistical power for the primary moderated mediation analysis, we conducted a post hoc power analysis using G^*^Power (Faul et al., 2007) for the moderating effect in a linear multiple regression model with 6 predictors. With α = 0.05, a medium effect size (*f*^2^ = 0.15), and our sample size of 1,218, the achieved power was 1.000, which substantially exceeds the conventional threshold of 0.80. Taken together, these data indicate that the sample size in this study is sufficient to provide adequate statistical power to test the hypothesized mediating, moderating, and conditional indirect effects. During the data preprocessing stage, the exclusion criteria included: (1) incomplete questionnaires (missing items ≥5%); (2) pattern-based responses (e.g., selecting the same option for more than 70% of the questions); (3) failure of the consistency test for reverse-scored items; (4) completion time less than 1 min (less than one-third of the average completion time from the pilot test). Based on the above criteria, a total of 182 invalid questionnaires were excluded, resulting in a final valid sample of 1,218 responses, with a valid response rate of 87.0%. The actual sample size exceeded the minimum requirement estimated a priori. The basic information of the final sample is as follows: In terms of gender distribution: 583 males (47.9%) and 635 females (52.1%); By academic year: 325 first-year undergraduates (26.7%), 320 second-year undergraduates (26.3%), 296 third-year undergraduates (24.3%), and 205 fourth-year undergraduates (16.8%); 72 graduate students and above (5.9%); By major: Humanities: 277 students (22.7%); Science and Engineering: 456 students (37.4%); Economics and Management: 228 students (18.7%); Arts and Physical Education: 195 students (16%); Medicine: 41 students (3.4%); Other: 21 students (1.7%).

### 2.2 Measurement instruments

#### 2.2.1 Outdoor sports atmosphere scale

The assessment used the Outdoor Sports Atmosphere Scale, developed by (Liu et al. 2011) for Chinese adolescents Liu et al., (2011). This scale assesses students' subjective perceptions of the outdoor sports atmosphere rather than objective environmental conditions. The scale consists of 17 items covering five dimensions: interpersonal connection, nature connection, interpersonal barriers, information access, and conditional barriers. It employs a 5-point Likert scale (1 = “Strongly Disagree” to 5 = “Strongly Agree”), with higher scores indicating a more positive perceived outdoor sports atmosphere. Among these, six items in the interpersonal barriers and environmental barriers dimensions—including “My parents do not encourage me to participate in outdoor sports,” “My school does not promote outdoor sports,” and “My academic workload is heavy, and I don't have time to participate in outdoor sports”—were reverse-scored items and were reversed prior to data analysis. In this study, the Cronbach's α coefficient for the questionnaire was 0.917. The results of confirmatory factor analysis showed: χ^2^*/df* = 1.620, GFI = 0.983, AGFI = 0.977, TLI = 0.993, CFI = 0.995, SRMR = 0.0212, and RMSEA = 0.023, indicating good structural validity of the scale.

#### 2.2.2 Core self-evaluation scale

The assessment used the Chinese version of the Core Self-Evaluation Scale (Du et al., 2012). The scale consists of 10 items scored on a 5-point Likert scale (1 = “Strongly Disagree” to 5 = “Strongly Agree”). Among these, six items—“I often feel down,” “I doubt my abilities,” “I feel that many things are terrible and hopeless,” are reverse-scored items; they were reversed before data analysis. Higher scores on all items indicate a higher level of core self-evaluation. In this study, the Cronbach's α coefficient for the questionnaire was 0.896. The results of confirmatory factor analysis showed: χ^2^*/df* = 2.306, GFI = 0.988, AGFI = 0.981, TLI = 0.992, CFI = 0.994, SRMR = 0.0193, and RMSEA = 0.033, indicating good structural validity for the scale.

#### 2.2.3 Digital health literacy scale

The assessment was conducted using the Chinese version of the eHealth Literacy Scale (Guo et al., 2013). The scale consists of 8 items, all of which are positively scored using a 5-point Likert scale (1 = “Strongly Disagree” to 5 = “Strongly Agree”); a higher score indicates a higher level of digital health literacy. In this study, the Cronbach's α coefficient for the questionnaire was 0.890. The results of confirmatory factor analysis showed: χ^2^*/df* = 3.876, GFI = 0.984, AGFI = 0.970, TLI = 0.983, CFI = 0.988, SRMR = 0.0178, and RMSEA = 0.049, indicating that the scale has good structural validity.

#### 2.2.4 Social-emotional competence scale

The assessment was conducted using the College Students' Social-Emotional Competence Scale (Chen Z. et al., 2023). Based on the CASEL social-emotional learning framework, this scale was localized to reflect the cultural characteristics of Chinese college students. It consists of 26 items covering four dimensions: self-relationship competence, other-relationship competence, group-relationship competence, and responsible decision-making competence. All items are positively scored using a 5-point Likert scale (1 = “Strongly Disagree” to 5 = “Strongly Agree”); higher scores indicate a higher level of social-emotional competence among college students. In this study, the Cronbach's α coefficient for the questionnaire was 0.936. The results of confirmatory factor analysis showed: χ^2^*/df* = 2.150, GFI = 0.960, AGFI = 0.952, TLI = 0.977, CFI = 0.980, SRMR = 0.0278, and RMSEA = 0.031, indicating good structural validity for the scale.

### 2.3 Data processing and analysis

This study used SPSS 27.0, AMOS 26.0, and the Process 4.1 SPSS macro plugin developed by Hayes for data organization and analysis. First, SPSS 27.0 was used to analyze the basic characteristics of the sample, conduct reliability tests, perform descriptive statistics, and conduct Pearson correlation analyses. Next, AMOS 26.0 was used to conduct confirmatory factor analysis and to explore predictive relationships among variables through regression analysis. Finally, Hayes' PROCESS macro (Models 4 and 7) was employed to analyze mediating and moderating roles: Model 4 tested the indirect effect of core self-evaluation on the relationship between the perceived outdoor sports atmosphere and social-emotional competence, while Model 7 tested whether digital health literacy moderates the indirect effect of core self-evaluation. All regression analyses controlled for gender, grade level, and academic major. These covariates were selected based on prior research indicating their potential associations with both environmental perceptions and psychosocial outcomes. Specifically, gender differences have been documented in peer relationship processes and social skills (Rose and Rudolph, 2006); grade level may reflect cumulative exposure to campus environments and developmental changes (Eccles and Roeser, 2011); and academic major may influence the types of social and physical activities students engage in. These demographic variables were controlled to reduce the risk of spurious associations and to isolate the unique effects of the primary predictors.

## 3 Results

### 3.1 Common method bias test

This study employed self-report questionnaires for data collection, with core variables assessed via participants' self-evaluations. This approach may introduce common method bias (CMB) due to single-source data, item wording, and design limitations. To evaluate the presence of CMB, we first conducted Harman's single-factor test. All items were subjected to unrotated exploratory factor analysis. The results extracted 11 factors with eigenvalues greater than 1, with the largest factor accounting for 25.42% of the variance, which did not exceed the 40% threshold (Podsakoff et al., 2003). This suggests that no single factor dominated the variance; however, Harman's test is a preliminary screening tool with limited sensitivity for detecting CMB and should not be used as the sole criterion (Podsakoff et al., 2012). Therefore, we further employed the unmeasured latent method construct (ULMC) approach for a more rigorous assessment. We constructed a confirmatory factor analysis model M1 (without a method factor) and a model M2 (with a common method factor) and compared their fit indices. M1 showed acceptable fit: χ^2^ = 2248.729, *df* = 1714, CFI = 0.987, TLI = 0.987, RMSEA = 0.016, AIC = 2602.729, BIC = 3506.308. M2 also showed acceptable fit: χ^2^ = 2152.127, df = 1653, CFI = 0.988, TLI = 0.987, RMSEA = 0.016, AIC = 2628.127, BIC = 3843.109. Although the chi-square difference was significant (Δχ^2^ = 96.60, Δ*df* = 61, *p* < 0.001), this test is sensitive to large sample sizes. More importantly, the changes in CFI (Δ = 0.001), TLI (Δ = 0.000), and RMSEA (Δ = 0.000) were all far below the conventional thresholds (ΔCFI < 0.01, ΔTLI < 0.01, ΔRMSEA < 0.015)(Cheung and Rensvold, 2002). In addition, both AIC and BIC increased in M2, indicating that adding the method factor did not improve model fit. These results suggest that common method variance is unlikely to represent a major threat to the observed associations; nevertheless, because these procedures have limited sensitivity, it cannot be entirely ruled out.

### 3.2 Descriptive statistics and correlation analysis

Prior to testing for mediating and moderating roles, this study conducted Pearson correlation analyses on each core variable to preliminarily examine the relationships among the variables. The results shown in (Table 1) indicate that the perceived outdoor sports atmosphere among college students is significantly positively correlated with their social-emotional competence (*r* = 0.385, *P* < 0.001), suggesting that the better the perceived outdoor sports atmosphere perceived by college students, the higher their level of social-emotional competence; the perceived outdoor sports atmosphere was significantly positively correlated with core self-evaluation (*r* = 0.393, *P* < 0.001) and with digital health literacy (*r* = 0.349, *P* < 0.001). Core self-evaluation was significantly positively correlated with social-emotional competence (*r* = 0.440, *P* < 0.001), and there was also a significant positive correlation between core self-evaluation and digital health literacy (*r* = 0.340, *P* < 0.001). Furthermore, digital health literacy was significantly positively correlated with social-emotional competence (*r* = 0.394, *P* < 0.001).

**Table 1:** Means, standard deviations, and correlation coefficients for each variable (*N* = 1218).

Variables	M	SD	OSA	CSE	DHL	SEC
OSA	3.356	0.761	1			
CSE	3.540	0.828	0.393^***^	1		
DHL	3.170	0.896	0.349^***^	0.340^***^	1	
SEC	3.509	0.705	0.385^***^	0.440^***^	0.394^***^	1

^*^p < 0.05, ^**^p < 0.01, ^***^p < 0.001; the same applies throughout. OSA, Outdoor Sports Atmosphere; CSE, Core Self-Evaluation; DHL, Digital Health Literacy; SEC, Social-Emotional Competence.

### 3.3 Analysis of the indirect effect of core self-evaluation

To test the indirect effect of core self-evaluation on the relationship between the outdoor sports atmosphere and social-emotional competence, this study employed the PROCESS macro (Model 4) developed by Hayes (2013) for analysis. The outdoor sports atmosphere was set as the independent variable, social-emotional competence as the dependent variable, and core self-evaluation as the mediating variable; gender, grade level, and major were included in the model as control variables. The Bootstrap method was used to perform 5,000 repeated samplings to calculate the 95% bias-corrected confidence interval for the indirect effect; if the confidence interval does not include 0, the indirect effect is considered significant. The specific regression analysis results (see Table 2) show that, after controlling for gender, grade level, and major, the perceived outdoor sports atmosphere was significantly positively associated with core self-evaluation (β = 0.428, *t* = 14.911, *p* < 0.001); When both the perceived outdoor sports atmosphere and core self-evaluation were included, core self-evaluation was significantly positively associated with social-emotional competence (β = 0.291, *t* = 12.573, *p* < 0.001); furthermore, the direct positive association of the perceived outdoor sports atmosphere on social-emotional competence remained significant (β = 0.232, *t* = 9.254, *p* < 0.001). The positive associations of the control variables (gender, grade level, major) were not significant in any of the models (*p* > 0.05). The results of the bootstrap mediation analysis (see Table 3) indicate that the indirect effect of core self-evaluation between the perceived outdoor sports atmosphere and social-emotional competence is 0.124, SE = 0.013, 95% CI [0.100, 0.150]; since this interval does not include 0, the mediation effect is significant and accounts for 34.73% of the total effect. The above results indicate that core self-evaluation showed a significant indirect association between the perceived outdoor sports atmosphere and social-emotional competence, thereby supporting Hypotheses H1 and H2.

**Table 2:** Results of the regression analysis for the core self-evaluation mediating model (*N* = 1,218).

Variables	Core self-evaluation				Social-emotional competence			
	β	***t***	**LLCI**	**ULCI**	β	***t***	**LLCI**	**ULCI**
gender	0.03	0.673	−0.057	0.116	0.036	1.005	−0.034	0.105
grade	−0.014	−0.759	−0.049	0.022	−0.013	−0.877	−0.042	0.016
major	−0.027	−1.478	−0.063	0.009	0.017	1.169	−0.012	0.047
OSA	0.428	14.911^***^	0.371	0.484	0.232	9.254^***^	0.183	0.282
CSE					0.291	12.573^***^	0.245	0.336
*R2*	0.157				0.248			
*F*	56.681^***^				80.079^***^			

^*^p < 0.05, ^**^p < 0.01, ^***^p < 0.001; All regression coefficients in this paper are unstandardized coefficients; the same applies throughout. LLCI denotes the lower limit of the 95% confidence interval; ULCI denotes the upper limit of the 95% confidence interval; the same applies throughout.

**Table 3:** Results of the bootstrap mediation analysis for core self-evaluation (*N* = 1218).

**Model effect**	Effect	BootSE	BootLLCI	BootULCI	Percentage of the Effect
Indirect effects	0.124	0.013	0.100	0.150	34.73%
Direct effect	0.232	0.025	0.183	0.282	65.27%
Overall effect	0.357	0.025	0.308	0.405	-

### 3.4 Analysis of the moderating role of digital health literacy

To examine the moderating role of digital health literacy, this study conducted a moderated mediation analysis using the PROCESS macro (Model 7) developed by Hayes, in which the perceived outdoor sports atmosphere served as the independent variable, core self-evaluation as the mediating variable, digital health literacy as the moderating variable, and social-emotional competence as the dependent variable. The results of the regression analysis (see Table 4) show that, after controlling for gender, grade level, and major, the interaction term between the perceived outdoor sports atmosphere and digital health literacy significantly predicted core self-evaluation (β = 0.135, *t* = 4.36, *p* < 0.001), indicating that digital health literacy significantly moderated the relationship between the perceived outdoor sports atmosphere and core self-evaluation.

**Table 4:** Analysis of the moderating role of digital health literacy (*N* = 1,218).

Variables	Core self-evaluation				Social-emotional competence			
	**β**	***t***	**LLCI**	**ULCI**	β	***t***	**LLCI**	**ULCI**
gender	0.029	0.691	−0.054	0.113	0.036	1.005	−0.034	0.105
grade	−0.01	−0.579	−0.045	0.024	−0.013	−0.877	−0.042	0.016
major	−0.029	−1.628	−0.064	0.006	0.017	1.169	−0.012	0.047
OSA	0.344	11.627^***^	0.286	0.401	0.232	9.254^***^	0.183	0.282
DHL	0.216	8.613^***^	0.167	0.265				
CSE					0.291	12.573^***^	0.245	0.336
OSA^*^DHL	0.135	4.36^***^	0.074	0.195				
*R2*	0.216				0.248			
*F*	55.756^***^				80.079^***^			

^*^p < 0.05, ^**^p < 0.01, ^***^p < 0.001; All continuous variables in the model were decentrized prior to analysis.

To further elucidate the moderating role of digital health literacy, this study conducted a simple slopes analysis (see Figure 2 and Table 5), dividing participants into high- and low-digital health literacy groups based on the mean plus or minus one standard deviation (M ± SD), and examined the positive association of the perceived outdoor sports atmosphere with core self-evaluation at different levels of digital health literacy. The results indicate that when digital health literacy levels were low (M−1SD), the perceived association of the perceived outdoor sports atmosphere with core self-evaluation was significant (Effect = 0.223, *t* = 5.586, 95% CI [0.145, 0.301]); when digital health literacy levels were high (M+1SD), the positive association was further enhanced and remained significant (Effect = 0.464, *t* = 11.311, 95% CI [0.384, 0.545]). These results indicate that digital health literacy positively moderates the relationship between the perceived outdoor sports atmosphere and core self-evaluation; that is, college students with high digital health literacy are better able to benefit from the perceived outdoor sports atmosphere to enhance their core self-evaluation. This supports Hypothesis H3.

**Figure 2:**
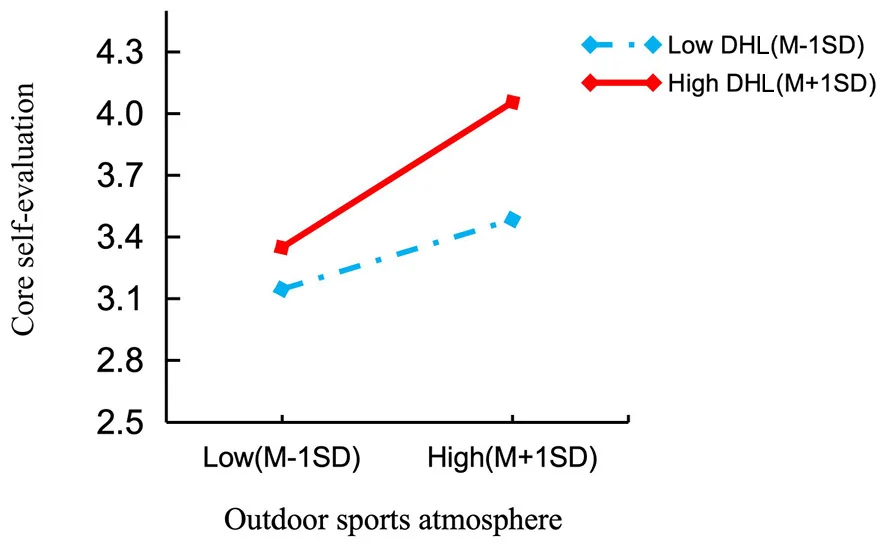
The moderating effect of digital health literacy on the relationship between outdoor sports atmosphere and core self-evaluation.

**Table 5:** Results of the simple slope analysis (*N* = 1,218).

**DHL**	Effect	se	*t*	LLCI	ULCI
(M−1SD)	0.223	0.04	5.586	0.145	0.301
(Mean)	0.344	0.03	11.627	0.286	0.401
(M+1SD)	0.464	0.041	11.311	0.384	0.545

This study further examined whether the indirect effect of core self-evaluation was moderated by digital health literacy. The results of the bootstrap test (see Table 6) showed that the Index of Moderated Mediation was 0.039 (95% CI [0.022, 0.058]), with the interval not containing zero, indicating that the moderated indirect effect was significant. Specifically, at low, medium, and high levels of digital health literacy, the indirect effect values of the perceived outdoor sports atmosphere on social-emotional competence via core self-evaluation were 0.065, 0.100, and 0.135, respectively, with none of the 95% Bootstrap confidence intervals containing zero. These results indicate that as digital health literacy levels increase, the indirect effect on social-emotional competence via core self-evaluation gradually strengthens, further confirming Hypothesis H4.

**Table 6:** Indirect effects and moderated mediation effect indices of core self-evaluation across different levels of digital health literacy.

DHL	Effect	BootSE	BootLLCI	BootULCI
(M−1SD)	0.065	0.012	0.042	0.088
(Mean)	0.100	0.011	0.079	0.122
(M+1SD)	0.135	0.015	0.106	0.166
Moderated mediating effect index	0.039	0.009	0.022	0.058

## 4 Discussion

This study examined the relationship between perceived outdoor sports atmosphere and social-emotional competence among college students, with core self-evaluation as a mediator and digital health literacy as a moderator of the first-stage pathway. Results indicated that perceived outdoor sports atmosphere was positively associated with social-emotional competence, both directly and indirectly through core self-evaluation. Digital health literacy moderated the association between perceived outdoor sports atmosphere and core self-evaluation, such that the indirect association was stronger at higher levels of digital health literacy.

### 4.1 The relationship between the perceived outdoor sports atmosphere and college students' social-emotional competence

This study found that perceived outdoor sports atmosphere was significantly positively associated with college students' social-emotional competence, consistent with social-ecological theory (Bronfenbrenner, 1979). As a microsystem, the school provides environmental resources—such as institutional support, peer norms, facility accessibility, and cultural encouragement—that may support students' emotional regulation and social interaction. Previous research has shown that school atmosphere is associated with social-emotional competence (Fang et al., 2025); this study extends this finding to the outdoor physical activity domain.

Specifically, this can be explained through the following pathways: First, nature-based recovery. According to the attention recovery theory (Kaplan, 1995), the natural environment can alleviate directed attention fatigue by eliciting unconscious attention, thereby providing cognitive resources for emotional regulation. Existing research indicates that outdoor activities such as hiking, cycling, and camping can significantly reduce anxiety and depression, improve mood, and strengthen social connections (Vasilaki et al., 2025); time spent playing outdoors is also positively associated with social-emotional competence (Ferguson et al., 2025). Second, interpersonal support. In outdoor sports settings, collaboration and encouragement among peers, along with teachers' positive guidance regarding participation, collectively create a supportive atmosphere for social interaction, providing students with opportunities to practice emotional expression, conflict resolution, and cooperative empathy in real-life situations. Existing research indicates that teachers' instrumental and emotional support (Fang and Tang, 2026), teacher care (Zhang et al., 2026), and positive teacher-student relationships (Li et al., 2026; Ma et al., 2026) can all effectively promote the development of students' social-emotional competencies. Third, information acquisition. Information acquisition in a perceived outdoor sports atmosphere refers to the guidance, feedback, and related media information received during participation in sports (Liu et al., 2011), which provides important cognitive resources for the development of social-emotional competencies. Existing research indicates that digital health literacy is significantly and positively correlated with both physical activity and social-emotional competencies (Zhao et al., 2026). Therefore, information acquisition serves as a crucial mediator linking the objective atmosphere to individual cognition within the perceived outdoor sports atmosphere; by enhancing the feasibility of participation and self-directed learning abilities, it provides cognitive support for the development of social-emotional competencies.

In summary, the perceived outdoor sports atmosphere is associated not only with higher levels of individuals' emotional regulation abilities through the restorative effects of the natural environment but is also related to social-emotional competencies through mechanisms of interpersonal support and information acquisition. This study focused on students' perceptions of the outdoor sports atmosphere rather than on objective environmental features. Perceptions are important in their own right, as they shape how individuals interpret and respond to their environment. The findings should therefore be interpreted as associations with perceived environmental support, rather than as direct evidence regarding actual campus facilities or resources.

### 4.2 The mediating role of core self-evaluation

The results indicate that core self-evaluation showed a significant indirect association between perceived outdoor sports atmosphere and social-emotional competence.

According to social cognitive theory (Bandura, 1986), the environment is indirectly related to an individual's psychology and behavior through their cognitive evaluation system. Perceived outdoor sports atmosphere may provide a framework for social comparison through behavioral references, such as peer norms and group values. These perceptions are associated with a more positive self-concept, which helps individuals overcome challenges and fosters a sense of self-confidence that is less dependent on external evaluations. These processes are consistent with evidence that exercise atmosphere influences exercise behavior through enjoyment and exercise self-efficacy as sequential mediators (Zhang et al., 2025a). In addition, interventions involving physical and mental exercise have been shown to reduce burnout and stress among female undergraduate students (Zhu et al., 2021), suggesting that structured physical activity experiences can help college students build psychological resources. As for the relationship between core self-evaluation and social-emotional competence, Judge et al. argue that individuals with higher core self-evaluation typically exhibit greater emotional stability, optimism, and interpersonal efficacy (Judge et al., 2003), and these traits are precisely the core components of social-emotional competence (Durlak et al., 2011). Existing research has confirmed that core self-evaluation is significantly and positively correlated with interpersonal adaptability (Zhang et al., 2025b) and social adaptability (Wang et al., 2025). However, these associations should be interpreted with caution given the cross-sectional design. Alternative directional pathways cannot be ruled out. For example, students with higher core self-evaluation or greater social-emotional competence may perceive their campus sports environment more positively or engage more actively in outdoor activities. This is consistent with Bandura's triadic reciprocal determinism, which emphasizes bidirectional interactions among personal, behavioral, and environmental factors (Bandura, 1986). It is worth noting that in this study, the indirect effect of core self-evaluation accounted for 34.73% of the total effect. This indicates that core self-evaluation is an important but not the sole explanatory pathway through which the perceived outdoor sports atmosphere is related to social-emotional competence; it may also indirectly affect social-emotional competence through other psychological mechanisms (such as emotional regulation, peer relationships, and self-efficacy), which future research may further explore.

### 4.3 The moderating role of digital health literacy

This study found that digital health literacy (DHL) positively moderates the relationship between perceived outdoor sports atmosphere and core self-evaluation; that is, the higher the level of DHL, the stronger the positive association between perceived outdoor sports atmosphere and core self-evaluation. However, DHL was assessed via self-report, reflecting a perceived ability rather than an objectively tested skill, and the specific digital behaviors discussed below were not directly assessed in this study. Therefore, the mechanisms proposed here should be regarded as possible theoretical explanations rather than empirically demonstrated pathways.

According to Resource Conservation Theory (Hobfoll, 2002), the prerequisite for the resource-gain spiral is that individuals possess a certain level of initial resources, which enables them to efficiently transform environmental resources into new psychological resources. Digital health literacy (DHL), as a typical form of initial information resource, essentially refers to an individual's ability to access, understand, evaluate, and apply health information (Norman and Skinner, 2006). It is theoretically plausible that students with higher DHL may utilize digital tools to locate outdoor facilities and to transform outdoor activities into trackable progress, thereby obtaining data-driven mastery feedback. This digitally enhanced sense of mastery resonates precisely with Bandura's assertion that “mastery experiences are the core source of self-efficacy” (Bandura, 1977) and further contributes to core self-evaluation (Judge et al., 2003). However, these specific behaviors were not directly measured in the present study. Conversely, students with low DHL may be prone to information overload when faced with abundant outdoor sports information due to insufficient filtering and integration strategies. This proposed cognitive mechanism also remains speculative, as cognitive load and information processing strategies were not directly measured. Furthermore, previous research has shown that DHL is significantly associated with healthy self-management behaviors, with self-efficacy playing a mediating role (Zhou Y. et al., 2025); DHL is also positively correlated with academic self-efficacy and online learning participation (Liu Y. et al., 2025). Li et al. found that DHL significantly moderates the relationship between exercise atmosphere and sleep quality (Li, 2024), while Prinitha and Jose discovered in a study of Indian adolescents that functional digital health literacy moderates the relationship between self-efficacy and HIV knowledge (Prinitha and Jose, 2025). These findings support the theoretical validity of DHL as a moderator, although they do not directly test the specific mechanisms proposed here. In summary, DHL is a cognitive-informational resource that primarily shapes how individuals perceive and interpret environmental information. This explains why DHL moderated the first stage of the mediation model—the link between perceived outdoor sports atmosphere and core self-evaluation—rather than the direct path to social-emotional competence. These findings suggest that higher digital health literacy may be associated with greater psychological benefits from a perceived outdoor sports atmosphere, although the specific mechanisms remain to be tested.

### 4.4 Limitations and future directions

This study explored the relationship between the perceived outdoor sports atmosphere and social-emotional competence, as well as its underlying mechanisms; however, several limitations remain that warrant further investigation. First, this study employed a cross-sectional design, with all variables measured at a single point in time. Therefore, the present findings should be interpreted as statistical associations consistent with the hypothesized model rather than as evidence of causal relationships, and bidirectional or even cyclical relationships among perceived outdoor sports atmosphere, core self-evaluation, and social-emotional competence cannot be ruled out. Future research could employ longitudinal tracking designs or experimental interventions to establish temporal precedence and causal directions. Second, the convenience sample from four universities in Chongqing limits generalizability; future research should use cross-regional, multi-center sampling to test contextual stability. In addition, although we controlled for gender, grade, and major, we did not measure other potential confounders (e.g., physical activity, socioeconomic status, health status), leaving residual confounding possible. Future research should include objective behavioral and socioeconomic or health measures to strengthen causal inference. Third, all variables were measured using self-report scales, and objective environmental indicators (e.g., actual facility availability, green-space coverage, institutional resources) were not assessed. Given the cross-sectional design, common method variance cannot be entirely ruled out, even though Harman's single-factor test and the ULMC analysis did not indicate a serious common method bias. Future research could combine subjective perceptions with objective environmental audits and incorporate multi-source data to reduce systematic errors and enhance the robustness of the conclusions. Fourth, this study examined only a single mediating variable: core self-evaluation. The pathways through which the perceived outdoor sports atmosphere is related to social-emotional competence may be more diverse. Future research could examine other potential mediating variables (such as emotion regulation, self-efficacy, peer relationships, and psychological resilience) or moderating variables (such as peer support, exercise motivation, and school physical education policies) to more comprehensively elucidate the psychosocial mechanisms through which the perceived outdoor sports atmosphere is related to social-emotional competence.

## 5 Conclusions

This study constructed a moderated mediation model to explore the relationship between the perceived outdoor sports atmosphere and college students' social-emotional competencies, as well as the underlying mechanisms. The results indicate that the perceived outdoor sports atmosphere is significantly positively correlated with social-emotional competencies; this association shows a direct association and an indirect association via core self-evaluation. Additionally, digital health literacy positively moderates the relationship between the perceived outdoor sports atmosphere and core self-evaluation. These findings suggest potential directions for future campus-based intervention research. Rather than focusing solely on promoting participation in outdoor physical activity or conveying mental health knowledge, future longitudinal or experimental studies could test whether interventions that combine environmental facilitation, cognitive shaping, and health-literacy development are effective in improving core self-evaluation and social-emotional competence.

## Data Availability

The raw data supporting the conclusions of this article will be made available by the authors, without undue reservation.
